# β2→1-Fructans Modulate the Immune System *In Vivo* in a Microbiota-Dependent and -Independent Fashion

**DOI:** 10.3389/fimmu.2017.00154

**Published:** 2017-02-16

**Authors:** Floris Fransen, Neha M. Sahasrabudhe, Marlies Elderman, Margaret Bosveld, Sahar El Aidy, Floor Hugenholtz, Theo Borghuis, Ben Kousemaker, Simon Winkel, Christa van der Gaast-de Jongh, Marien I. de Jonge, Mark V. Boekschoten, Hauke Smidt, Henk A. Schols, Paul de Vos

**Affiliations:** ^1^Top Institute Food and Nutrition, Wageningen, Netherlands; ^2^Department of Pathology and Medical Biology, University Medical Center Groningen, University of Groningen, Groningen, Netherlands; ^3^Laboratory of Food Chemistry, Wageningen University, Wageningen, Netherlands; ^4^Microbial Physiology, Groningen Biomolecular Sciences and Biotechnology Institute (GBB), University of Groningen, Groningen, Netherlands; ^5^Laboratory of Microbiology, Wageningen University, Wageningen, Netherlands; ^6^Laboratory of Pediatric Infectious Diseases, Radboud University Medical Center, Nijmegen, Netherlands; ^7^Nutrition, Metabolism and Genomics Group, Division of Human Nutrition, Wageningen University, Wageningen, Netherlands

**Keywords:** β2→1-fructans, prebiotics, gut microbiota, mucosal immunology, germ-free mice

## Abstract

It has been shown *in vitro* that only specific dietary fibers contribute to immunity, but studies *in vivo* are not conclusive. Here, we investigated degree of polymerization (DP) dependent effects of β2→1-fructans on immunity *via* microbiota-dependent and -independent effects. To this end, conventional or germ-free mice received short- or long-chain β2→1-fructan for 5 days. Immune cell populations in the spleen, mesenteric lymph nodes (MLNs), and Peyer’s patches (PPs) were analyzed with flow cytometry, genome-wide gene expression in the ileum was measured with microarray, and gut microbiota composition was analyzed with 16S rRNA sequencing of fecal samples. We found that β2→1-fructans modulated immunity by both microbiota and microbiota-independent effects. Moreover, effects were dependent on the chain-length of the β2→1-fructans type polymer. Both short- and long-chain β2→1-fructans enhanced T-helper 1 cells in PPs, whereas only short-chain β2→1-fructans increased regulatory T cells and CD11b^−^CD103^−^ dendritic cells (DCs) in the MLN. A common feature after short- and long-chain β2→1-fructan treatment was enhanced 2-alpha-l-fucosyltransferase 2 expression and other IL-22-dependent genes in the ileum of conventional mice. These effects were not associated with shifts in gut microbiota composition, or altered production of short-chain fatty acids. Both short- and long-chain β2→1-fructans also induced immune effects in germ-free animals, demonstrating direct effect independent from the gut microbiota. Also, these effects were dependent on the chain-length of the β2→1-fructans. Short-chain β2→1-fructan induced lower CD80 expression by CD11b^−^CD103^−^ DCs in PPs, whereas long-chain β2→1-fructan specifically modulated B cell responses in germ-free mice. In conclusion, support of immunity is determined by the chemical structure of β2→1-fructans and is partially microbiota independent.

## Introduction

The intestinal tract contains the largest pool of immune cells in the body, which are separated by only a single layer of epithelial cells from an enormous amount of environmental stimuli, including the gut microbiota and dietary components (1). *Via* a complex of regulatory mechanisms, our immune system manages to be tolerant against food antigens and commensal microorganisms, yet a strong immune response can be raised against pathogens. The balance between tolerance and inflammatory responses is delicate and disturbances are implicated in an ever growing list of Western diseases, such as inflammatory bowel disease, allergies, diabetes, and even cancer (2). Therefore, modulation of the intestinal immune system with for example dietary fibers is considered an attractive strategy to prevent or treat these diseases (3).

Among the different dietary fibers, inulin β2→1-fructans have been studied extensively (4). β2→1-fructans are a heterogeneous group of polysaccharides, which can be found in several plants, but are most often commercially obtained from chicory (4). These fibers consist of several fructose subunits connected *via* β2→1 linkages, with or without a terminal glucose molecule (5). Chain-length of β2→1-fructans can be described by the degree of polymerization (DP), which ranges from 2 to 60, referring to the number of monomers that are part of the polymeric structure. Shorter polymers with a DP < 10 are often referred to as fructo-oligosaccharide (FOS), whereas longer polymers with DP > 10 are usually called inulin.

β2→1-fructans have been shown to modulate the immune system (6). For example, short-chain β2→1-fructans have been demonstrated to increase IgA secretion and production of IL-10 and interferon-γ by CD4^+^ T cells from Peyer’s patches (PPs) in mice (7, 8), whereas long-chain β2→1-fructans induced higher numbers of dendritic cells in PPs and greater *ex vivo* secretion of IL-2, IL-10, and interferon-γ from spleen and mesenteric lymph node (MLN) cells after administration to rats (9). Generally, these immune effects have been attributed to the ability of β2→1-fructans to promote the expansion of immunostimulatory bacteria in the gut, mainly Bifidobacteria and Lactobacilli (10). In addition, metabolites produced after fermentation of β2→1-fructans by commensal gut bacteria include short-chain fatty acids (SCFAs), which dampen inflammatory responses by binding to G protein-coupled receptors GPR41, GPR43, and GPR109A expressed on different types of immune cells (11–14). Recently, we also demonstrated that β2→1-fructans can stimulate immune cells directly *in vitro via* activating pattern recognition receptors such as toll-like receptors (TLRs) (15). This direct effect on immune cells *in vitro* is stronger with higher DP β2→1-fructans than with shorter DP β2→1-fructans. Thus, several possible mechanisms by which β2→1-fructans can modulate the immune system have been proposed, but the existence or contribution to immune effects *in vivo* is not clearly studied yet. Also, it has not been shown yet *in vivo* that immune modulation of β2→1-fructans is DP dependent. This knowledge is essential as it might lead to selection of effective immune modulating dietary fibers helpful in the arms race between dietary fiber intake and Western diseases (16).

Here, we performed an *in vivo* study in mice with well-defined short and long-chain β2→1-fructans to determine (1) whether β2→1-fructans are able to modulate the immune system *in vivo*, (2) whether there are differences between short- and long-chain β2→1-fructans with respect to the type of immune responses that are induced and (3) the direct effects of β2→1-fructans on the immune system.

## Materials and Methods

### Mice

Specified pathogen free conventional C57BL/6OlaHsd males of 8 weeks old were purchased from a commercial supplier (Envigo, Horst, the Netherlands), and C57BL/6OlaHsd male 8 weeks old germ-free mice were obtained from a breeding colony at the animal facility of the Radboud University (Nijmegen, the Netherlands). Samples derived from germ-free mice were routinely screened once every 2 weeks on sterility. In addition, samples from germ-free mice the day before the end of the experiment were tested on sterility. All samples were screened on sterility by QM diagnostics (Nijmegen, the Netherlands) and confirmed to be sterile. At least 2 weeks before the start of β2→1-fructans treatment all animals were receiving a D12450B diet (10% fat, Research Diet Services, Wijk bij Duurstede, the Netherlands). The mice were kept on this diet throughout the experiment. All experiments were approved by the animal ethics committee of the University of Groningen (Dierenexperimentencommissie Rijksuniversiteit Groningen, DEC-RUG).

### β2→1-Fructans

Frutalose^®^OFP is a highly soluble powdered short-chain β2→1-fructan also called FOS of mainly DP2-10, produced by partial hydrolysis of chicory inulin. Frutafit^®^TEX! (both obtained from Sensus, Roosendaal, the Netherlands), is a powdered food ingredient of DP10-60, based on native chicory inulin. DP profiles of supplement A and B are depicted in Figure S1 in Supplementary Material.

Endotoxin levels were tested by Toxikon (Leuven, Belgium) and found to be below 0.3 × 10^−3^ µg^−1^. The fructans were dissolved in sterile water and given to the conventional mice by oral gavage. In addition, to ensure sterility of these solutions before administration to germ-free mice, short-chain β2→1-fructans were filtered with a 0.2 µm filter and long-chain β2→1-fructans were irradiated with 25 kGy (Synergy Health, Ede, the Netherlands), as it was to viscous for sterilization by filtration. Irridation induced some degradation of higher DP oligomers, but still significant amounts of oligomers up to DP30 were present. Sterility of the fructans after filtration or irradiation was tested by QM diagnostics (Nijmegen, the Netherlands) and confirmed to be sterile. Mice received 10 mg of these fibers or water only as a negative control by oral gavage once a day for 5 days in a row.

### High-Performance Anion-Exchange Chromatography (HPAEC)

Colonic content from each mouse was first dissolved in PBS (100 mg/ml) and the supernatant was stored at −20°C after separation from the debris by centrifugation. These samples together with cecum contents derived from the same mice as prepared in the same way were used to analyze for short- and long-chain β2→1-fructans with HPAEC, which was performed on an ICS5000 system (Thermo Fisher Scientific, Waltham, MA, USA), equipped with a Dionex CarboPac PA-1 column (2 mm × 250 mm) in combination with a Carbopac PA-1 guard column (2 mm × 50 mm). Ten microliters of the samples were injected into the system using a Thermo Fischer Scientific ICS5000 autosampler. The system was equipped with pulsed amperometric detection (ED40 gold electrode). The flow rate was 0.3 ml/min, and the gradient used was 0–5 min 0.1 M NaOH, from 5 min in 40 min to 0.4 M NaOAc in 0.1 M NaOH; 5 min isocratically at 1 M NaOac in 0.1 M NaOH finally an equilibration step for 15 min at starting conditions. The software used was Chromeleon version 7 (Dionex). Monosugars and maltodextrin were used as standards.

### High-Performance Liquid Chromatography (HPLC)

For SCFA analysis of cecum content, HPLC was performed to quantify butyric acid, propionic acid, acetic acid, lactic acid, and succinic acid on a Ultimate 3000 HPLC (Thermo Fisher Scientific, Waltham, MA, USA) equipped with an autosampler, a RI-101 refractive index detector (Shodex, Kawasaki, Japan), and an ion-exclusion Aminex HPX 8H column (7.8 mm × 300 mm) with a guard column (Bio-Rad, Hercules, CA, USA). The mobile phase was 5 mM H_2_SO_4_, and the flow rate was 0.6 ml/min at 65°C. Samples were injected onto the column and SCFA production was quantified.

### Flow Cytometry

Spleen, PPs, and MLNs were isolated from each mouse. PPs were removed from the small intestine by tightening the patch with tweezers and cutting it with scissors, avoiding as much as possible inclusion of the surrounding intestinal epithelium. Fat tissue was carefully removed from the MLNs with a razor blade. After isolation, all organs were immediately placed in RPMI, 10% FBS, 1% Pen/Strep on ice. Single cell suspensions were obtained from all organs by crushing the organs with the plunger of a 2.5-ml syringe on 70 µm nylon cell strainers placed over a 50-ml tube. Red blood cells in spleen samples were lysed with a hypotonic lysis buffer. Cells were stained with Fixable Viability Dye eFluor 50 (eBioscience, Vienna, Austria) to exclude dead cells. Aspecific binding to FC receptors was prevented by incubating the cells with anti-CD16/32 (clone 93, Biolegend, Uithoorn, the Netherlands) for 15 min on ice. For extracellular staining, cells were incubated with the desired mixture of antibodies for 30 min on ice. After washing, cells were fixed with FACS lysing solution (BD Biosciences, Breda, the Netherlands). For intracellular staining, fixed cells were permeabilized with PERM (eBioscience, Vienna, Austria) and subsequently stained with the desired antibodies for 30 min on ice. For identification of the different T helper cell subsets, cells were stained with antibodies against: CD3e (clone 17A2), CD4 (clone GK1.5), T-bet (clone 4B10), RORyt (clone B2D), Gata-3 (clone TWAJ), CD25 (clone PC61), and Foxp3 (clone FJK-16S). Appropriate isotype controls were used to determine specificity of the staining. To identify naive or antigen-experienced T cells, the following antibodies were used: CD3e (clone 17A2), CD4 (clone GK1.5), CD8a (clone 53-6.7), CD69 (clone H1.2F3), CD44 (clone IM7), and CD62L (clone MEL-14). B-cells were stained with: CD19 (clone 6D5), B220 (clone RA-6B2), IgD (clone RTK2758), IgM (clone II/41), IgA (clone mA-6E1). For analysis of dendritic cells (DCs), other lineages were first excluded with CD3e (clone 17A2), CD19 (clone 6D5), B220 (clone RA-6B2), and NK1.1 (clone PK136). Next, DC subsets were identified with CD11c (clone HL3), MHC-II (clone M5/114.15.2), CD11b (clone M1/70), CD103 (clone 2E7), CD8a (clone 53-6.7), and CD80 (clone 16-10 A1). Samples were acquired with the FACSVerse (BD Biosciences, Breda, the Netherlands) and analyzed with Flow Jo software (Flow Jo LLC, OR, USA).

### Transcriptomics

A part of the terminal ileum from each mouse was snap frozen in liquid nitrogen and stored at −80°C. RNA was isolated with the RNeasy kit (Qiagen, Valencia, CA, USA). Quantity of RNA was measured with the ND-1000 (NanoDrop Technologies, Thermo Fisher Scientific, Breda, the Netherlands) and quality of RNA was assessed with the Bioanalyzer 2100 (Agilent, Santa Clara, CA, USA). Total RNA (100 ng) was labeled utilizing the Ambion WT Expression kit (Life Technologies Ltd., Bleiswijk, the Netherlands) and the Affymetrix GeneChip WT Terminal Labeling kit (Affymetrix, Santa Clara, CA, USA). After labeling, samples were hybridized to Affymetrix GeneChip Mouse Gene 1.1 ST arrays. An Affymetrix GeneTitan Instrument was used for hybridization, washing, and scanning of the array plates. Bioconductor packages integrated in an online pipeline were used for quality control of the data (17, 18). Probe sets were redefined using current genome information (19). Probes were reorganized based on the Entrez Gene database (remapped CDF v14.1.1). Robust Multiarray Analysis preprocessing algorithm available in the Bioconductor library affyPLM (20) was used to obtain normalized expression estimates from the raw intensity values.

### Fluorescence *In Situ* Hybridization

Another part of ileum and colon was fixed in Carnoy’s fixative to preserve the mucus layer. Samples were embedded in paraffin and afterward 4 µm sections of tissue were made with a microtome (Leica Biosystems, Nussloch, Germany). Paraffin was removed from the tissue slides by washing 2× with xylene for 10 min. Next, samples were washed in decreasing amounts of ethanol (99, 70, 60%) and finally in dH_2_O. After washing, samples were incubated with 0.5 µg universal bacterial probe EUB338 (5′-GCTGCCTCCCGTAGGAGT-3′) or segmented filamentous bacteria (SFB)-specifc probe SFB1008 (5′-GCGAGCTTCCCTCATTACAAGG-3′) conjugated on the 5′ end with Alexa 488 (Eurogentec, Maastricht, the Netherlands) in hybridization solution (20 mM TRIS-HCl, pH 7.4, 0.9 M NaCl, 0.1% weight/volume SDS) at 50°C overnight in a humid environment. The next day, the samples were washed in FISH washing buffer (20 mM TRIS-HCl, pH 7.4, 0.9 M NaCl) for 20 min. Tissue slides were then washed 2× for 10 min with PBS and incubated with DAPI solution (30 nM) for 5 min to visualize nuclei of the epithelial cells. After washing 2× with PBS, samples were mounted with mounting medium. Data were acquired with a SP8 Leica confocal microscope (Leica Microsystems, Son, the Netherlands), and images were processed afterward with Imaris software (Bitplane, Zurich, Switzerland).

### Microbiota Analysis

Fresh fecal samples obtained just after defecation were collected from all conventional mice before the start of fiber treatment. In addition, colonic content samples from these mice were collected at the end of the experiment. All samples were snap frozen in liquid nitrogen and stored at −80°C. These samples were used for 16S rRNA gene analysis for microbiota profiling with barcoded amplicons from the V1–V2 region of 16S rRNA genes generated using a 2-step PCR strategy that reduces the impact of barcoded primers on the outcome of microbial profiling (21). DNA extraction was performed using a combination of the bead-beating-plus column method and the Maxwell 16 Tissue LEV Total RNA purification kit (Promega, Leiden, the Netherlands). Beating of the fecal pellets took place as described before (22) but with STAR (Stool transport and recovery) buffer (Roche, Basel Switzerland). After centrifugation, 250 µl supernatant was taken for the Maxwell 16 Tissue LEV Total RNA Purification Kit, and the DNA was eluted in 50 µl DNAse free water. Twenty nanograms of DNA were used for the amplification of the 16S rRNA gene with primers 27F-DegS and 338R I + 338R II for 25 cycles as described before (23), only primers had a Universal Tag (UniTag) linkers attached; UniTag I (forward) and II (reverses) (I—GAGCCGTAGCCAGTCTGC; II—GCCGTGACCGTGACATCG). The first PCR was performed in a total volume of 50 µl containing 1× HF buffer (Finnzymes, Vantaa, Finland), 1 µl dNTP Mix (10 mM; Promega, Leiden, the Netherlands), 1 U of Phusion^®^ Hot Start II High-Fidelity DNA polymerase (Finnzymes Vantaa, Finland), 500 nM of the 27F-DegS primer (23, 24) that was appended with UniTag 1 at the 5′ end, 500 nM of an equimolar mix of two reverse primers, 338R I and II (24) based on three previously published probes EUB 338 I, II, and III (23), that were 5′-extended with UniTag 2, and 0.2–0.4 ng/µl of template DNA. The sequence of the UniTags were selected to have a GC content of ~66% and a minimal tendency to form secondary structures, including hairpin loops, heterodimers, and homodimers as assessed by the IDTDNA Oligoanalyzer 3.1 (Integrated DNA Technologies). Moreover, sequences were selected that had no matches in 16S rRNA gene databases [based on results of the “TestProbe” tool offered by the SILVA rRNA database project (25) using the SSU r117 database], and no prefect matches in genome databases with the Primer-BLAST tool (http://www.ncbi.nlm.nih.gov/tools/primer-blast/). The size of the PCR products (~375 bp) was confirmed by gel electrophoresis using 5 µl of the amplification reaction mixture on a 1% (w/v) agarose gel containing 1× SYBR^®^ Safe (Invitrogen, Thermo Fisher Scientific, Waltham, MA, USA). Five microliters of these PCR products were taken to add adaptors and a 8-nt sample-specific barcode in an additional five cycle PCR amplification. This second PCR was performed in a total volume of 100 µl containing 1× HF buffer, dNTP Mix 2 U of Phusion^®^ Hot Start II High-Fidelity DNA polymerase, 500 nM of a forward and reverse primer equivalent to the Unitag 1 and UniTag 2 sequences, respectively, that were each appended with an 8 nt sample-specific barcode (Hermes et al., in preparation) at the 5′ end. PCR products were purified with the magnetic beads (MagBio, London, UK) according to the HighPrepTM protocol of the manufactures instructions using 20 µl Nuclease Free Water (Promega Leiden, the Netherlands) and quantified using the Qubit (Life Technologies, Bleiswijk, the Netherlands). Purified PCR products were mixed in approximately equimolar amounts and concentrated by the magnetic beads as the purification before. Purified amplicon pools were 250 bp paired-end sequenced using Illumina Miseq (GATC-Biotech, Konstanz, Germany).

The Illumina Miseq data analysis was carried out with a workflow employing the Quantitative Insights Into Microbial Ecology (QIIME) pipeline (26) and a set of in-house scripts as described before for Illumina Hiseq 16S rRNA gene sequences (Hermes et al., in preparation). The set of in-house scripts processed the reads as follows: reads were filtered for not matching barcodes; otu picking and chimera removal was done *via* matching the sequences to the Silva 111 database, with only one mismatch allowed, and a biom and with clustalw a multiple alignment and phylogenetic tree file was generated. Further outputs were generated *via* QIIME, such as filtered reads per sample, PD whole tree diversity measurements and the level 1–6 taxonomic distributions with relative abundances.

### Statistics

Flow cytometry data are expressed as means, error bars represent SEM. To verify whether there were significant differences between the groups, one way ANOVA was performed followed by the Dunnett’s multiple comparisons test. All tests were performed with Graphpad software (Prism, La Jolla, CA, USA).

Differentially expressed probe sets were identified using linear models, applying moderated T-statistics that implemented empirical Bayes regularization of SEs (27). A Bayesian hierarchical model was used to define an intensity-based moderated T-statistic, which takes into account the degree of independence of variances relative to the degree of identity and the relationship between variance and signal intensity (28).

Statistical tests for gut microbiota composition were performed using R and Calypso (29), where the count data were not normally distributed and variances between groups were not equal, the Mann–Whitney *U* test was used.

## Results

### β2→1-Fructans Modulate the Mucosal Immune System *In Vivo*

We demonstrated previously that inulin β2→1-fructans stimulate immune cells directly and in a DP-dependent manner *in vitro* (15). In this *in vivo* study in mice, the immune effects of chemically characterized short (DP2-10) and long-chain chain β2→1-fructans (DP10-60) were compared. To this end, conventional mice received these fibers by oral gavage once a day for 5 days. Effects were studied in the PPs, MLNs, and spleen.

**Figure 1:**
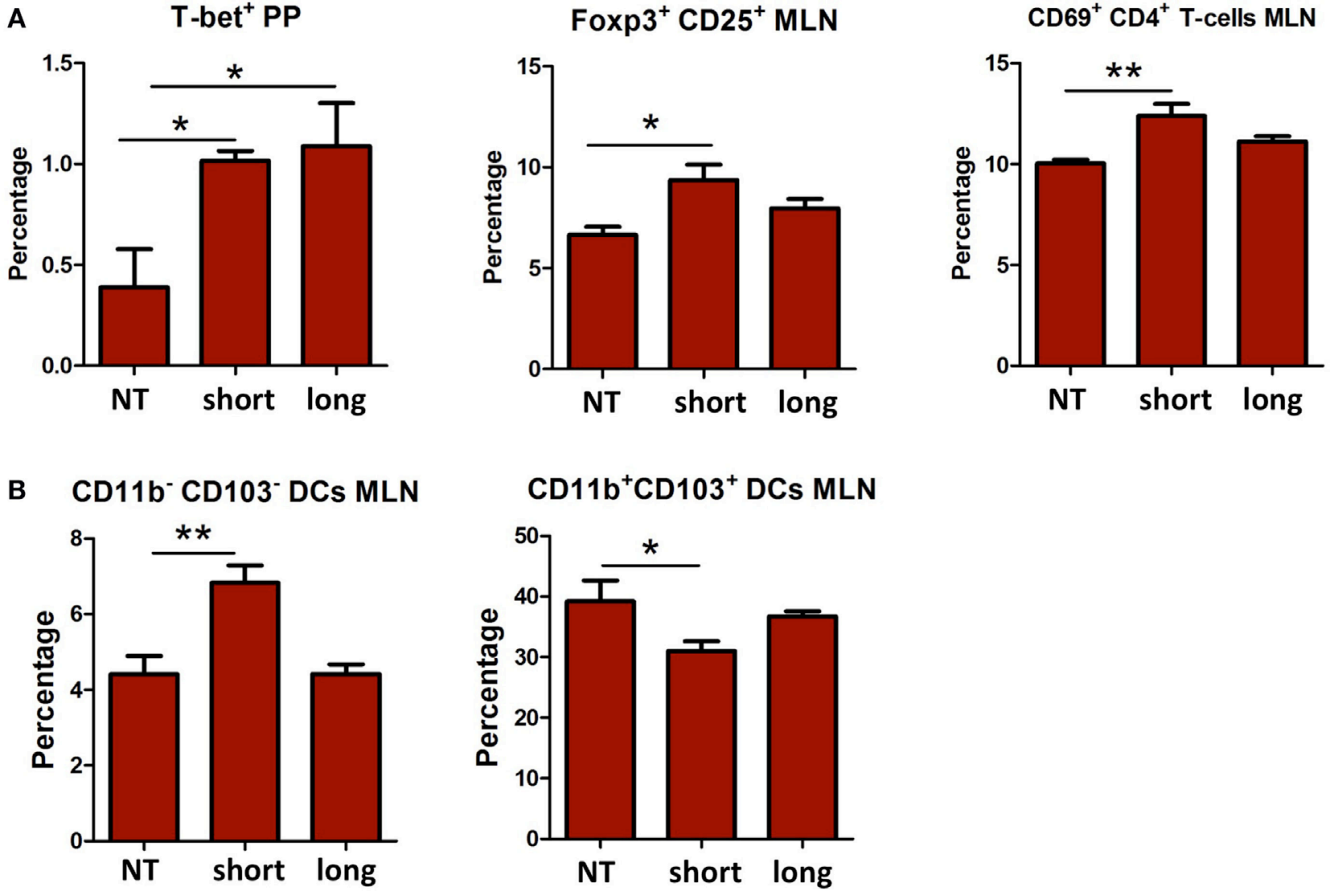
**β2→1-fructans modulate immune responses *in vivo***. C57BL/6 mice (*n* = 5 per group) received short-chain β2→1-fructans (short), long-chain β2→1-fructans (long), or water (NT) for five constitutive days by oral gavage. Spleen, Peyer’s patches (PPs), and mesenteric lymph nodes (MLNs) were isolated for FACS analysis of immune cell populations. **(A)** Percentages of Th1 cells (T-bet^+^) Tregs cells (CD25^+^Foxp3^+^), and CD69^+^ cells among CD3e^+^CD4^+^ cells **(B)** CD11c^hi^MHC-II^hi^ cells negative for the lineage markers CD3e, CD19, B220, and NK1.1 were divided in the four major dendritic cell subsets with CD11b and CD103. Only data are shown for the organs where there was a significant difference after β2→1-fructan treatment. All data are expressed as means, error bars represent SEM (*p < 0.05, **p < 0.01).

Th1, Th2, Th17, and Treg cells in the different organs were compared between mice treated with the two β2→1-fructans and controls (Figure 1A). No differences were found in the spleen (data not shown). However, in the PPs, both short and long-chain β2→1-fructan induced a higher number of Th1 cells (*p* < 0.05) compared to controls. In addition, in MLNs, short-chain β2→1-fructan but not long-chain β2→1-fructan treatment resulted in higher percentages of Treg cells (*p* < 0.05) and CD4^+^ T cells expressing the activation marker CD69 (*p* < 0.05). Next, we assessed the influence of β2→1-fructan treatment on B-cells in these tissues. No differences were observed in B cells in any organ tested (data not shown). Finally, considering the important role of DCs in initiating and modulating adaptive immune responses, we also evaluated the effect of β2→1-fructan treatment on DCs (Figure 1B). Strikingly, mice treated with short-chain but not the mice treated with long-chain β2→1-fructan had an increase in CD11b^−^CD103^−^ DCs in the MLNs (*p* < 0.01) and a decrease in CD11b^+^CD103^+^ DCs (*p* < 0.05). No differences were found in spleen or PPs (data not shown).

Thus, β2→1-fructans can modulate the immune system locally in the PP and MLN *in vivo* in a DP-dependent fashion. Only short-chain β2→1-fructan modulated DC and Treg numbers in MLNs.

### β2→1-Fructans Induce Chain-Length Dependent Changes in Gene Expression in the Ileum

**Figure 2:**
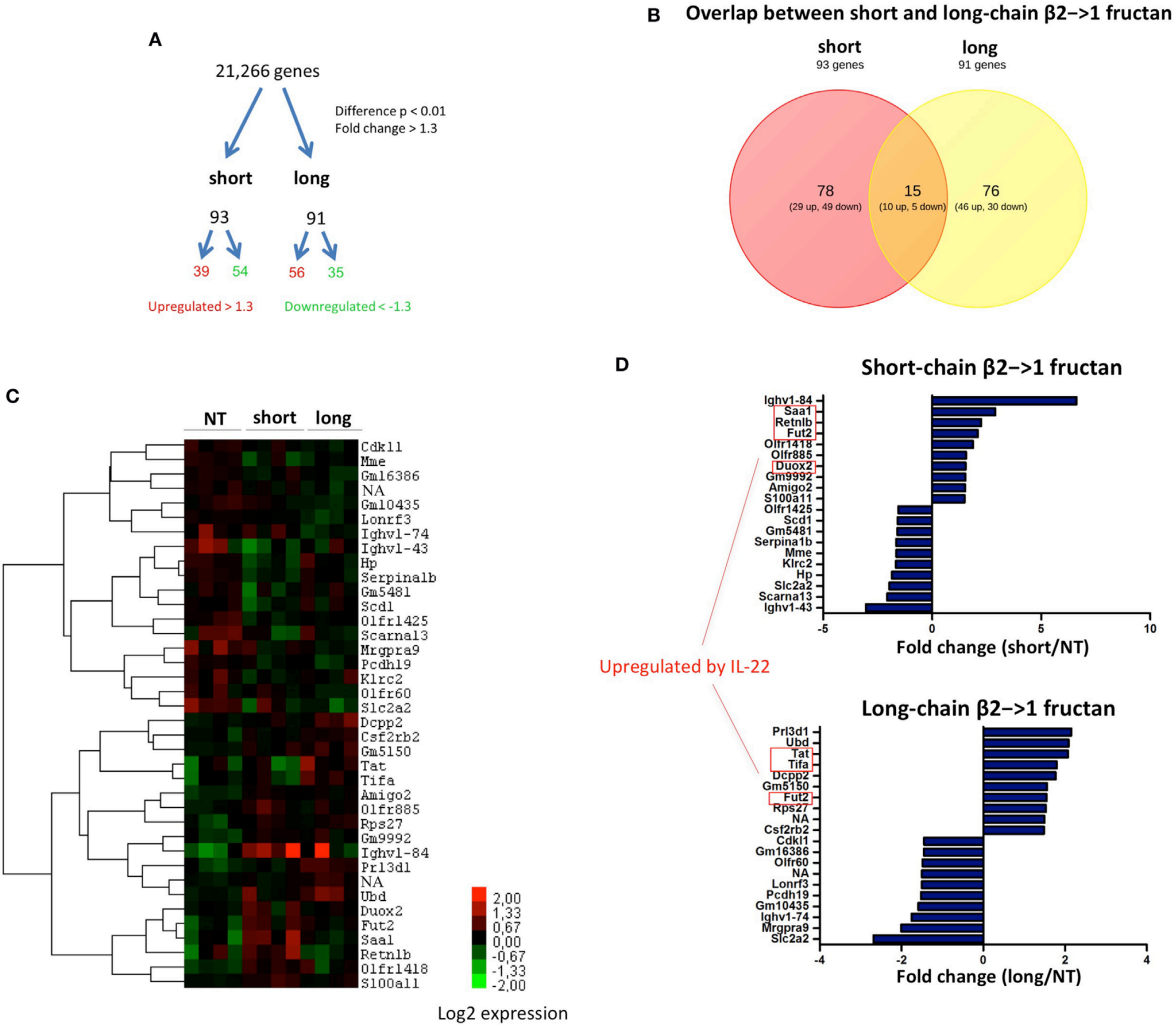
**Differential gene expression in the ileum after β2→1-fructan treatment**. Whole-genome gene expression in the ileum of C57BL/6 mice (*n* = 4 per group) treated with short-chain β2→1-fructans (short), long-chain β2→1-fructans (long), or water was assessed with Affymetrix GeneChip Mouse Gene 1.1 ST arrays. **(A)** Number of genes that were considerably differentially expressed (*p* < 0.01, fold-change > 1.3) after β2→1-fructan treatment compared to controls. **(B)** Overlap in the number of genes differentially expressed after treatment with short-chain β2→1-fructans or long-chain β2→1-fructans. **(C)** Hierarchical clustering heatmap for each individual mouse of the 40 genes that were most highly upregulated or downregulated after treatment with short-chain β2→1-fructans or long-chain β2→1-fructans. **(D)** Gene expression relative to controls of the 10 most highly upregulated or downregulated genes after treatment with short-chain β2→1-fructans or long-chain β2→1-fructans. Genes previously shown to be induced by IL-22 are indicated (31).

To investigate the different impact in an unbiased manner, effects of the two β2→1-fructans on the host whole-genome expression of ileal tissue were studied with microarray. The ileum is assumed to be the location where β2→1-fructans interact with the mucosa (6). Treatment with both short and long-chain β2→1-fructan induced significant differential gene expression of approximately 100 genes in the ileum (*p*-value < 0.01, fold-change > 1.3) compared to controls (Figure 2A). Remarkably, the majority of affected genes were uniquely and differently regulated by either short- or long-chain β2→1-fructans (Figures 2B,C). There was only a minimal overlap in the induced responses by these β2→1-fructans. One of these genes was galactoside 2-alpha-l-fucosyltransferase 2 (Fut2), an enzyme involved in glycosylation. It was among the top-10 most upregulated genes by both short- and long-chain β2→1-fructans (Figure 2D). Recently, it has been demonstrated that Fut2 is upregulated by microbiota-dependent stimulation of IL-22 production by type 3 innate lymphoid cells, which in turn stimulates colonization resistance against pathogens (30). That IL-22 is regulated by both short- and long-chain β2→1-fructans was further confirmed by the observation that (i) genes known to be upregulated in epithelial cells by IL-22 (31) were strongly enriched in the top-10 most upregulated genes (Figure 2D) and (ii) that several of these genes clustered together after hierarchical cluster analysis (Figure 2C). However, short-chain β2→1-fructan seemed to induce a stronger response, reflected by higher expression of microbiota-dependent genes such as Fut2, SAA1, Retnlb, and Duox2 (Figures 2C,D). As upregulation of the IL-22-Fut2 axis is assumed to be gut microbiota dependent (30), these results suggest that the effects of β2→1-fructans on gene expression in the ileum are caused by differences in effects on gut microbiota. Therefore, we next studied and compared the microbiota composition in the mice treated with short- and long-chain β2→1-fructans.

### Short- and Long-Chain β2→1-Fructans Did Not Alter Gut Microbiota Composition

**Figure 3:**
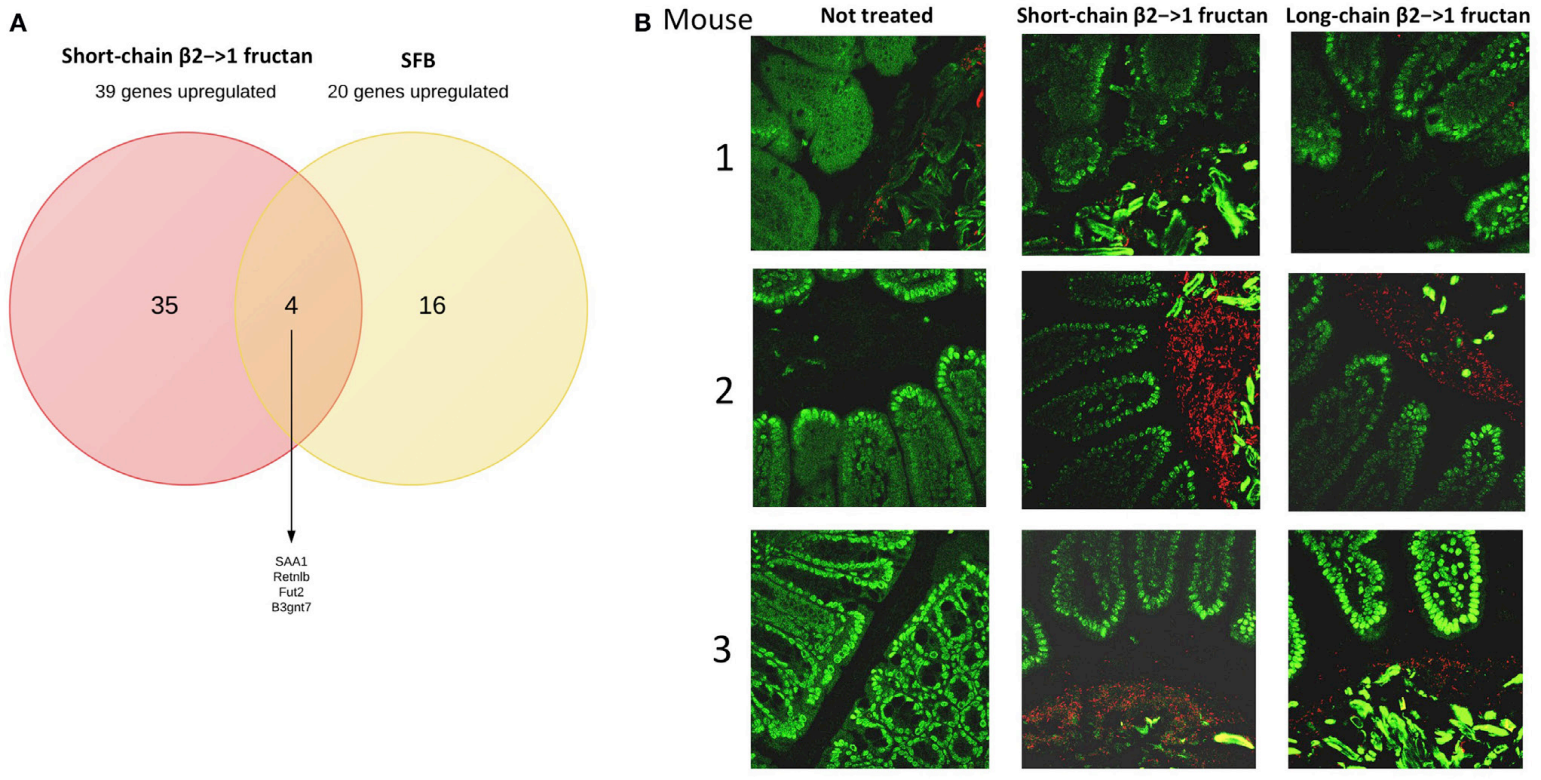
**β2→1-fructans stimulate expansion of bacteria in ileum**. **(A)** Overlap between the 39 most highly upregulated genes in the ileum by short-chain β2→1-fructans and the previously reported top-20 most upregulated genes (32) after segmented filamentous bacteria colonization. **(B)** Ileum sections of mice (*n* = 3 per group) treated with short-chain β2→1-fructans, long-chain β2→1-fructans, or water (not treated) were stained with the universal bacterial probe EUB338 (red) and DAPI (green) to visualize the gut microbiota and nuclei of epithelial cells, respectively.

As SFB promote production of IL-22 and upregulation of Fut2 (30), we first studied the ileum of short- and long-chain β2→1-fructan treated animals for possible differences in colonization by SFB (Figure 3A). Although we observed an overall higher density of microbiota after FISH staining (Figure 3B), especially in animals receiving short-chain β2→1-fructans, we never found SFB in the ileum in any sample. Also by applying 16S rDNA sequencing in fecal samples, we excluded promotion of SFB in the β2→1-fructans-treated mice (data not shown), which suggest that other mechanisms must be responsible for FUT2 upregulation.

**Figure 4:**
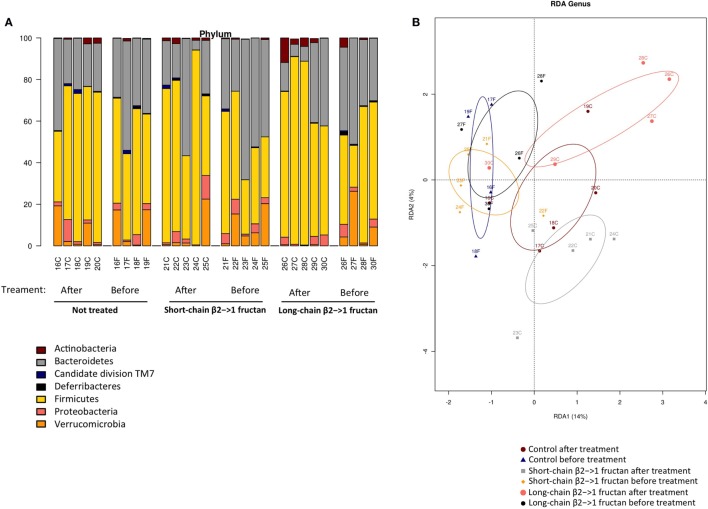
**β2→1-fructan treatment does not alter gut microbiota composition**. Fecal samples (*n* = 4–5 per group) were collected before or after treatment with short-chain β2→1-fructans, long-chain β2→1-fructans, or water (not treated/control). Bacterial DNA was isolated and analyzed with 16S rDNA sequencing. Numbers indicate individual mice before or after treatment, indicated with “F” and “C,” respectively. **(A)** Distribution of the different bacterial groups at the phylum level for all samples. **(B)** Redundancy analysis (RDA) for all samples at the genus level.

Since β2→1-fructans have been shown to modulate gut microbiota composition (10), we analyzed gut microbiota composition with 16S rDNA sequencing in fecal samples before and after treatment with the dietary fibers (Figure 4A). No significant differences were found in any specific bacterial group after treatment (data not shown). Moreover, in agreement with the SFB-specific FISH staining, SFB was not detected in any of the samples (data not shown). Redundancy analysis suggested that gut microbiota composition was similar between the groups before treatment, as expected (Figure 4B). The samples from the three groups posttreatment separated into three different clusters, suggesting that β2→1-fructans had some effect on the composition of the microbial community, but this separation was not significant (Figure 4B).

### β2→1-Fructans Are Degraded in the Gut But Do Not Induce Enhanced SCFA Production

**Figure 5:**
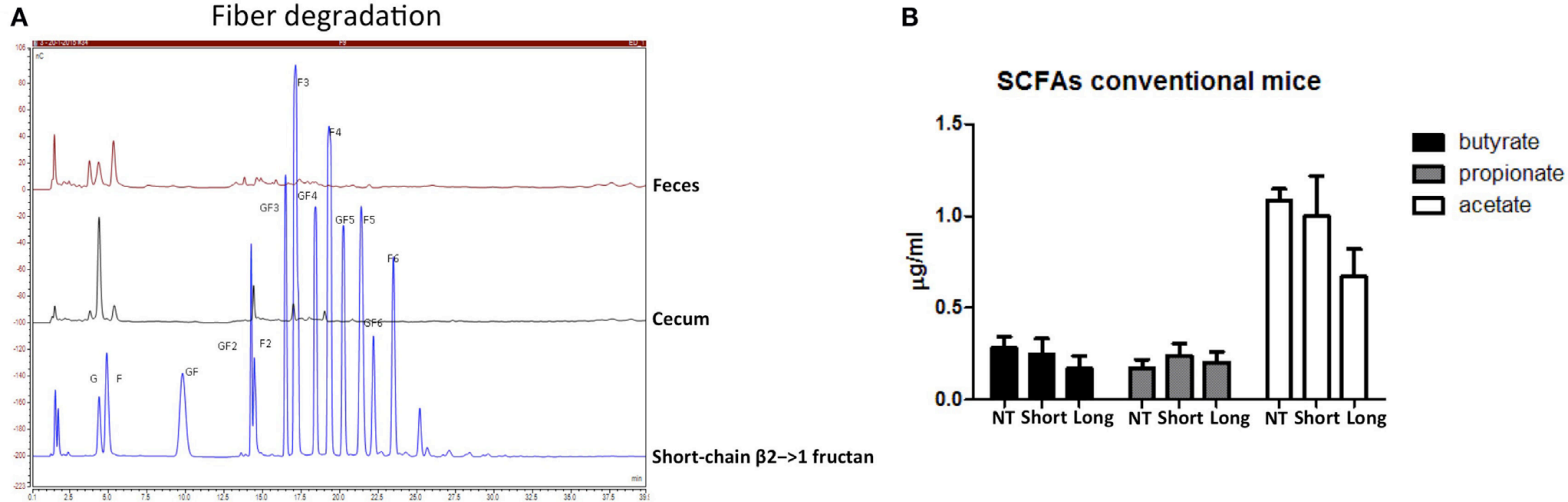
**β2→1-fructans are degraded in the gut but do not increase short-chain fatty acid (SCFA) levels**. **(A)** Colonic and cecal content of mice that received short-chain β2→1-fructans was analyzed with high-performance anion-exchange chromatography and compared to short-chain β2→1-fructan. **(B)** Cecal samples from mice (*n* = 5 per group) that received short-chain β2→1-fructans (short), long-chain β2→1-fructans (long), or water (NT) were analyzed with high-performance liquid chromatography to quantify butyric acid, propionic acid, acetic acid, lactic acid, and succinic acid. Lactic acid and succinic acid were not detected (data not shown).

Next, despite the absence of differences in microbiota composition between short- and long-chain β2→1-fructan-treated mice, we studied possible differences in immune active SCFA production as explanation for the observed immune differences. However, concentrations of butyrate, propionate, acetate, lactic acid, and succinic acid in cecal samples were not different, nor enhanced by the two β2→1-fructans (Figure 5B). As it might be suggested that this might be caused by lack of fermentation of the β2→1-fructans, we quantified the fructans in the colon and cecum of β2→1-fructan-treated mice by HPAEC. Both β2→1-fructans were undetectable, indicating completely utilization by the gut microbiota (Figure 5A, only plot of short-chain fructan is shown).

### β2→1-Fructans Modulate Immune Responses in Germ-Free Mice

As we could not find pronounced difference in short and long-chain β2→1-fructans induced effects on microbiota or its degradation products, we determined and compared the impact of the two β2→1-fructans on the mucosal immunity in germ-free mice.

**Figure 6:**
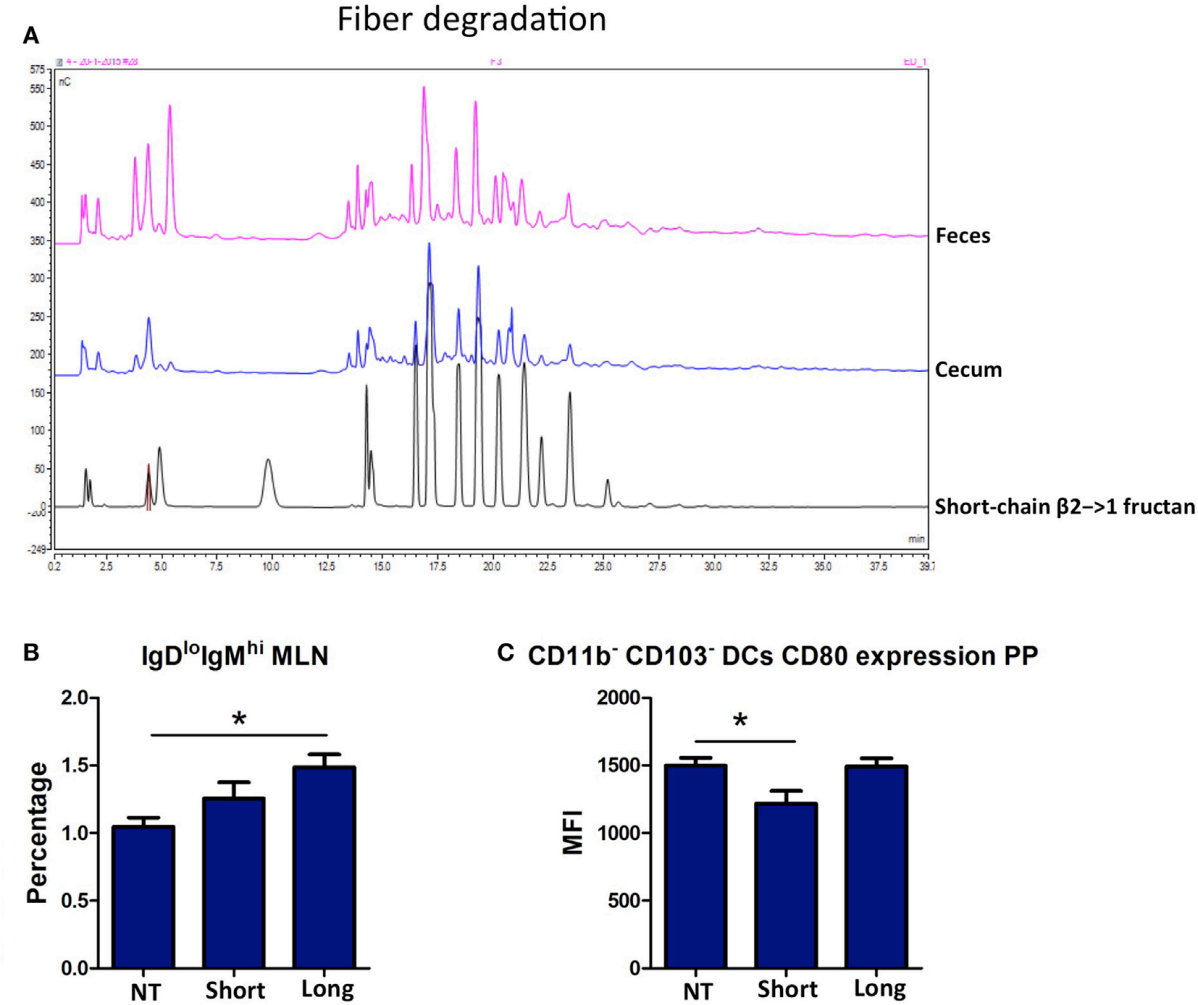
**β2→1-fructans modulate the immune system in germ-free mice**. **(A)** Colonic and cecal content derived from germ-free mice that received short-chain β2→1-fructans was analyzed with high-performance anion-exchange chromatography and compared to short-chain β2→1-fructan. **(B)** Percentage of IgD^lo^IgM^hi^ cells among CD19^+^B220^+^ B-cells in the mesenteric lymph nodes of mice (*n* = 5 per group) after receiving short-chain β2→1-fructans (short), long-chain β2→1-fructans (long), or water (NT). In addition, in the Peyer’s patches (PPs) of theses mice CD80 expression as measured by the median fluorescence intensity (MFI) is shown for CD11b-CD103-dendritic cells **(C)**. Data are expressed as means, error bars represent SEM, and *p < 0.05.

As expected, β2→1-fructans were not degraded in the intestine of germ-free mice (Figure 6A), and no SCFAs could be detected (data not shown). Analysis of immune cells population in the PPs, MLNs, and spleens was performed in the same fashion as in conventional mice. Th1, Th2, Th17, and Treg cells were not influenced by β2→1-fructans in the absence of microbiota (data not shown). In contrast, we did observe effects on B-cells and DCs in β2→1-fructan-treated germ-free mice, and the effects were DP dependent. Long-chain β2→1-fructans induced enhanced numbers of IgD^lo^IgM^hi^ B-cells in the MLNs (*p* < 0.05, Figure 6B) but not in the PPs or spleen (data not shown), while short-chains did not have such an effect. DCs in PPs were only modulated by short-chain β2→1-fructans as expression of the activation marker CD80 was lower in PP DCs that did not express CD11b or CD103 (*p* < 0.05) (Figure 6C). Only graphs of regulated cells are shown.

### Microbiota-Independent β2→1-Fructan-Induced Changes in Gene Expression in the Ileum

**Figure 7:**
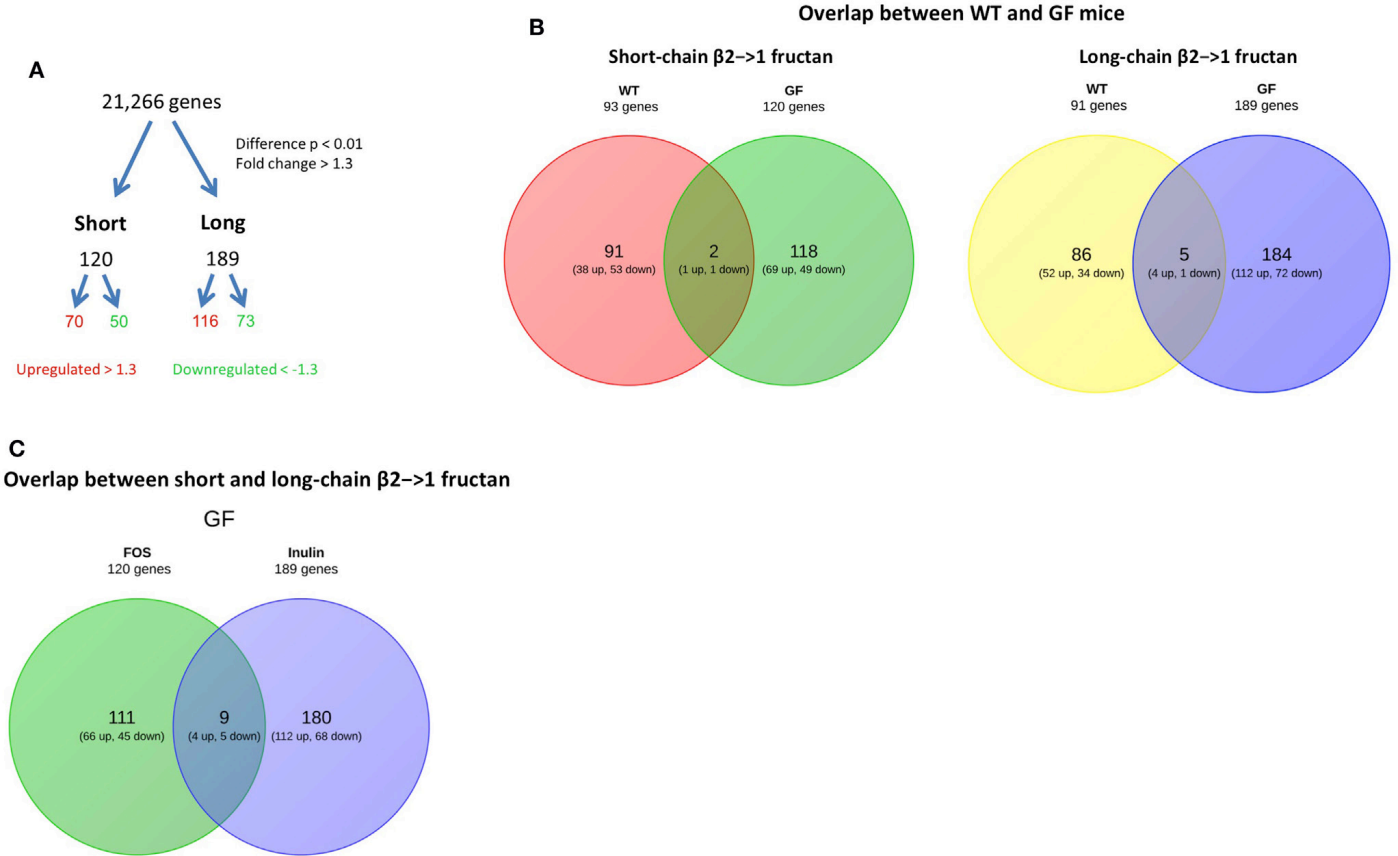
**β2→1-fructans induce differential gene expression in the ileum**. Whole-genome gene expression in the ileum of germ-free mice (*n* = 4 per group) treated with short-chain β2→1-fructans (short), long-chain β2→1-fructans (long), or water was assessed with Affymetrix GeneChip Mouse Gene 1.1 ST arrays. **(A)** Number of genes that were considerably differentially expressed (*p* < 0.01, fold-change > 1.3) after β2→1-fructan treatment compared to controls. **(B)** Overlap between conventional and germ-free mice in the number of genes differentially expressed after treatment with short-chain β2→1-fructans or long-chain β2→1-fructans. **(C)** Overlap in the number of genes differentially expressed in germ-free mice between short-chain β2→1-fructans and long-chain β2→1-fructans.

Also, the ileum of β2→1-fructan treated germ-free mice was studied by whole-genome expression with microarray. We found that both short- and long-chain β2→1-fructans modulate the gene expression in the ileum in the absence of the gut microbiota (Figure 7A). First, the gene profiles of short-chain β2→1-fructan-treated germ-free animals were compared to that of treated mice with a conventional microbiome to determine unique microbiota-dependent and microbiota-independent effects. The same was done for long-chain β2→1-fructan. After that, we compared the DP-dependent effects in the germ-free animals.

As shown in Figure 7B, there was only minor overlap between the genes affected in conventional and germ-free mice for both short and long-chain fructans, suggesting that β2→1-fructans modulate different pathways by direct interaction and by microbiota-dependent effects.

Strikingly, when studying DP-dependent effects in the mice there was also minimal overlap between differential gene expression induced by short and the long-chain treated germ-free mice (Figure 7C). This illustrates that similarly to what we observed in conventional mice, chain-length of β2→1-fructans is key in determining the type of induced response.

### Long-Chain β2→1-Fructan Modulates B-Cell Response in Germ-Free Mice

**Figure 8:**
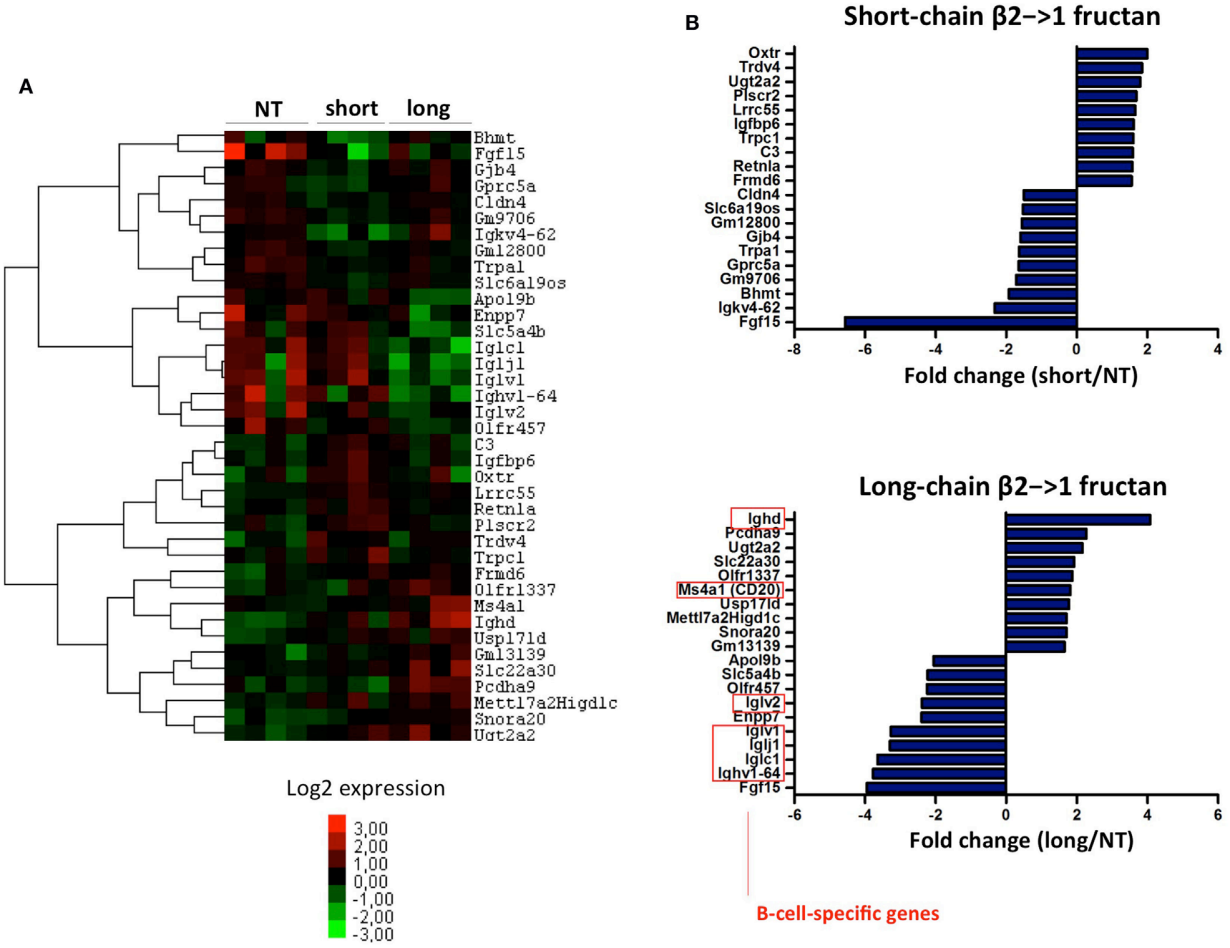
**Long-chain β2→1-fructans modulate B-cell responses**. **(A)** Hierarchical clustering heatmap for each individual germ-free mouse of the 40 genes that were most highly upregulated or downregulated after treatment with short-chain β2→1-fructans (short) or long-chain β2→1-fructans (long) compared to controls (NT). **(B)** Gene expression relative to controls (NT) of the 10 most highly upregulated or downregulated genes after treatment with short-chain β2→1-fructans (short) or long-chain β2→1-fructans (long). Affected genes after long-chain β2→1-fructan treatment that are specifically expressed by B-cells are indicated.

A heatmap of the 40 most differentially expressed genes in the ileum of germ-free mice after treatment with short or irradiated long-chain inulin further confirmed the presence of gene clusters specifically modulated by either short- and long-chain β2→1-fructan (Figure 8A). In particular, long-chain β2→1-fructan seemed to modulate B-cell responses, since several genes involved in antibody production were differentially expressed, including a strong upregulation of IgD and CD20 (Figure 8B). These data correspond to the flow cytometry data, where we observed an increase in IgD^lo^IgM^hi^ B-cells in the MLNs of germ-free mice after inulin treatment.

## Discussion

The structure–effector relationship between dietary fibers and health benefits *in vivo* is an area that deserves further exploration. Differences in immunomodulation by non-digestible dietary fibers is generally considered to depend on differences in stimulation of immunomodulatory bacteria and/or their fermentation products such as SCFA (33). Several studies have shown that a diet with a high content of non-digestible fiber leads to higher levels of SCFAs in the intestine (34). However, we did not observe any differences in SCFA levels after fructan treatment. Therefore, it seems unlikely that the observed effects are mediated by SCFAs. Possibly 5 days of treatment is too short to enhance SCFA production.

We recently demonstrated *in vitro* that the dietary fibers β2→1-fructans can activate immune cells directly in a chain-length dependent manner (15). Here, we demonstrate to the best of our knowledge for the first time that direct effects of dietary fibers on immunity also occur *in vivo*. Our germ-free mice study confirms that direct effects of β2→1-fructans are also *in vivo* chain-length dependent.

One of the major findings of our study is that the chain-length of β2→1-fructans is crucial in dictating the type of immune response *in vivo*. In conventional mice both fibers enhanced the number of Th1 cells in the PPs, but only short-chain β2→1-fructan treatment increased the number of Tregs and CD11b^−^CD103^−^ DCs in the MLNs. Therefore, our data seem to suggest that in response to short-chain β2→1-fructans CD11b^−^CD103^−^ DCs migrate from the LP to the MLN to induce Tregs. Also, the whole-genome gene expression data indicate stronger immune responses in the ileum after short-chain β2→1-fructan treatment compared to long-chain β2→1-fructans. These differences might reflect the fact that short-chain β2→1-fructans are fermented earlier and more readily in the gastro-intestinal tract than long-chain β2→1-fructans due to the shorter polymeric structure (35, 36). The highest concentration of immune cells can be found in the ileum, where the mucus layer is much thinner compared to the colon (1). Several bacterial species that occupy this niche are known to interact with the immune system (37). Targeting these bacteria with relatively easily digestible short-chain β2→1-fructans might therefore be an effective strategy for modulation of immune responses.

A second, and very important finding is the demonstration of effects of β2→1-fructans on the immune system in the absence of the microbiota by using germ-free mice. In these mice, we observed a clear difference between short- and long-chain β2→1-fructan. These differences could be due to differential direct interactions with pattern recognition receptors such as TLRs on immune cells, as described previously (15). Long-chain β2→1-fructans, but not short-chain β2→1-fructans, induced strong modulation of B-cell responses in germ-free mice. Long-chain β2→1-fructans have been shown to activate TLR2 more strongly *in vitro* (15) and could have activated TLRs expressed on B-cells more strongly than for short-chain fructans *in vivo*. TLR ligands are well known for their ability to activate B-cells (38). Alternatively, long-chain β2→1-fructans could have activated B-cells in antigen-specific manner in germ-free mice. Presumably, 5 days of treatment with long-chain β2→1-fructans would be too short to detect specific antibodies. However, we did observe upregulation of IgD and CD20 in the ileum in germ-free mice treated with long-chain β2→1-fructans, which may indicate expansion of antigen-specific B-cells, before class-switching to another isotype such as IgA.

To the best of our knowledge, we demonstrate for the first time that β2→1-fructan treatment can enhance Fut2 expression, which is an enzyme involved in fucosylation of epithelial cells in the gut. This effect was observed with both DPs. Fut2 was recently shown to be induced by microbiota-dependent (especially SFB) stimulation of IL-22 production by type 3 innate lymphoid cells, which promoted colonization resistance against mucosal pathogens (30, 31, 39). Here, we observed that several genes such as Fut2, SAA1, Retnlb, and Duox2 that were previously shown to be induced by IL-22 (31) were highly upregulated in the ileum after treatment with β2→1-fructans. Fut2 induced upregulation by β2→1-fructans may therefore be a novel mechanism by which these dietary fibers contribute to improved colonization of bacteria and reported health effects (6).

Although we excluded that SFB colonization was responsible for Fut2 expression, we do not exclude that β2→1-fructans enhanced other gut microbes that induced Fut2 expression. Other commensals, such as *Bacteroides*, have been shown to induce epithelial fucosylation as well (40, 41). However, we did not find a significant shift in gut microbiota composition in fecal samples after β2→1-fructan treatment. However, FISH staining of ileum samples suggests higher number of bacteria in the ileum after β2→1-fructan treatment. Therefore, it remains possible that β2→1-fructans induced local expansion of specific bacterial species in the small intestine, which subsequently enhanced expression of Fut2 in the epithelium.

In humans, application of β2→1-fructans to induce upregulation of Fut2 for enhancement of resistance against mucosal pathogens (30, 31, 39) might be more complex than in mice. Approximately 20% of the human population lacks a functional copy of Fut2, which are referred to as non-secretors (42). The role of Fut2 in development of several diseases has been demonstrated by comparing secretor and non-secretors. The Fut2 non-secretors have a different microbiota composition with less *Bifidobacteria* (43) and are less susceptible to enteric viruses (44), but are more susceptible to Crohn’s disease (45). Fut2 manipulation with dietary fibers such as β2→1-fructans may therefore be an interesting approach to alleviate symptoms or frequency of intestinal diseases in the 80% of secretors.

In conclusion, our data suggest that rational design of β2→1-fructans formulations with different chain-lengths is a promising and probably a necessary approach to achieve the desired effect in different target populations. We demonstrate that effects can be both microbiota dependent and independent. Moreover, although dietary fibers are considered as beneficial for health in general, our data demonstrate that at least in case of β2→1-fructans this depends on the chain length of the fiber.

## Ethics Statement

This study was carried out in accordance with the recommendations of FELESA guidelines and the ethical committee for animal experiments from the University of Groningen (DEC-RUG). The protocol was approved by the he ethical committee for animal experiments from the University of Groningen (DEC-RUG).

## Author Contributions

FF and PV designed experiments and wrote the manuscript. FF, NS, ME, MB, FH, TB, BK, and SW performed experiments. SA, FH, and HS analyzed microbiota composition data. MB and HAS provided and analyzed HPAEC and HPLC data. MVB generated and analyzed microarray data. CJ and MJ provided reagents and resources. PV supervised the project.

## Conflict of Interest Statement

The authors declare that the research was conducted in the absence of any commercial or financial relationships that could be construed as a potential conflict of interest.

## Supplementary Material

Figure S1 **Degree of polymerization (DP) distribution in short- and long-chain β2→1-fructan**. DP distribution in short-chain β2→1-fructan (short) and long-chain β2→1-fructan.

Figure S2 **FACS plots T cells. Gating strategy and representative FACS plots (mesenteric lymph nodes sample) for identifying T cell subsets**. **(A)** Among CD3^+^CD4^+^ T cells the percentage of Tregs (Foxp3^+^CD25^+^), Th1 cells (T-bet^+^), Th17 cells (RORgt^+^), and Th2 cells (GATA-3^+^) was identified. Percentage of cells stained with isotype control was subtracted to determine the percentage of true positive cells. **(B)** Percentages of naïve (CD44^lo^CD62L^hi^), memory cells (CD44^hi^CD62L^hi^), and effector cells (CD44^hi^CD62L^lo^) among CD3^+^CD4^+^ and CD3^+^CD8^+^ cells. In addition, the percentage of CD69^+^ cells was identified among CD3^+^CD4^+^ and CD3^+^CD8^+^ cells.

Figure S3 **FACS plots B cells and dendritic cells (DCs)**. Gating strategy and representative FACS plots (mesenteric lymph nodes sample) for identifying B cell and DC subsets. **(A)** B cells were identified as CD19^+^B220^+^ and further divided in IgD^hi^IgM^lo^, IgD^hi^IgM^hi^, or IgD^lo^IgM^hi^. In addition, IgA-producing B cells were identified as IgD^−^IgA^+^ **(B)** DCs were identified as lineage negative (CD3^−^CD19^−^B220^−^NK1.1^−^) and CD11c^hi^MHC-II^hi^. These cells were further divided into four subsets based on the expression of CD11b and CD103. In addition, CD80 expression was assessed for each DC subset.

Figure S4 **Segmented filamentous bacteria (SFB) FISH staining**. A representative image (one mouse treated with short-chain β2→1-fructans) of ileum samples fixed in Carnoy’s fixative that were stained with SFB-specifc probe SFB1008 conjugated to Alexa Fluor 488 (green) and DAPI (blue). Some green spots were identified, but they lacked the typical morphology of SFB.
