# Supplementary figures and images for: β2→1-Fructans Modulate the Immune System In Vivo in a Microbiota-Dependent and -Independent Fashion
Source: Front Immunol. 2017 Feb 16;8:154. doi: 10.3389/fimmu.2017.00154 (PMC5311052; doi:10.3389/fimmu.2017.00154)

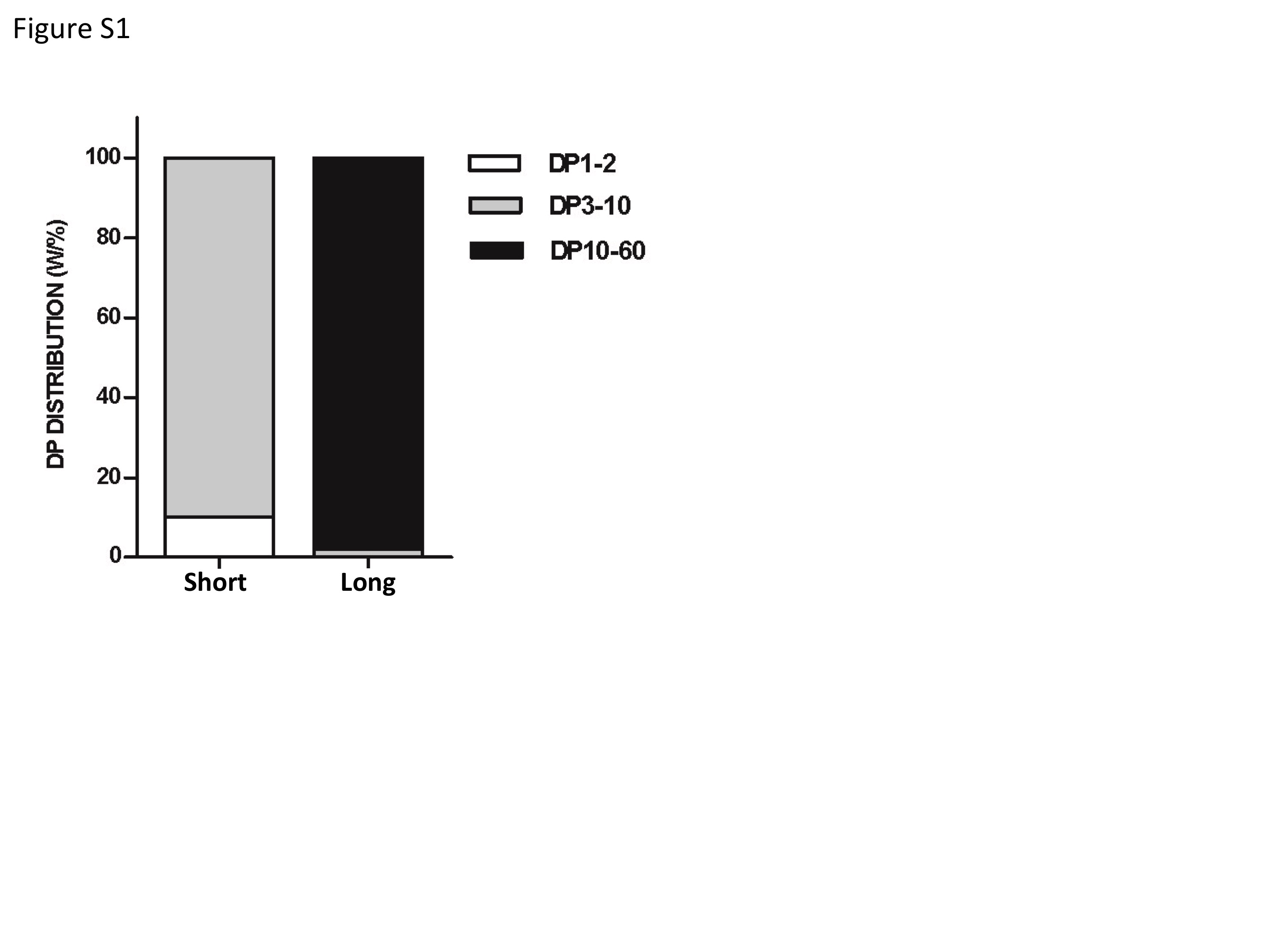

Supplement: Figure S1 — Degree of polymerization (DP) distribution in short- and long-chain β2→1-fructan. DP distribution in short-chain β2→1-fructan (short) and long-chain β2→1-fructan. [file image_1.jpeg]

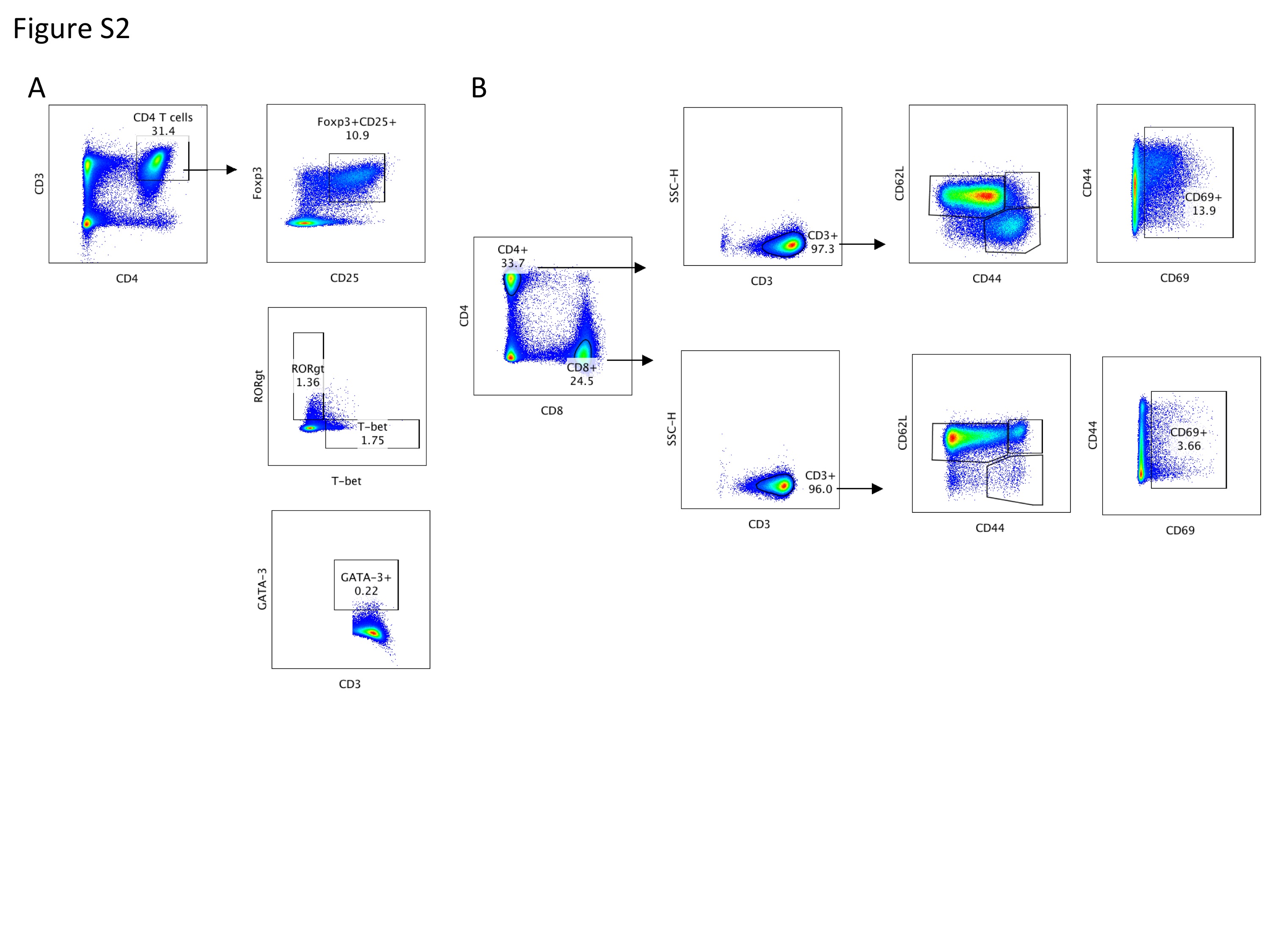

Supplement: Figure S2 — FACS plots T cells. Gating strategy and representative FACS plots (mesenteric lymph nodes sample) for identifying T cell subsets. (A) Among CD3+CD4+ T cells the percentage of Tregs (Foxp3+CD25+), Th1 cells (T-bet+), Th17 cells (RORgt+), and Th2 cells (GATA-3+) was identified. Percentage of cells stained with isotype control was subtracted to determine the percentage of true positive cells. (B) Percentages of naïve (CD44loCD62Lhi), memory cells (CD44hiCD62Lhi), and effector cells (CD44hiCD62Llo) among CD3+CD4+ and CD3+CD8+ cells. In addition, the percentage of CD69+ cells was identified among CD3+CD4+ and CD3+CD8+ cells. [file image_2.jpeg]

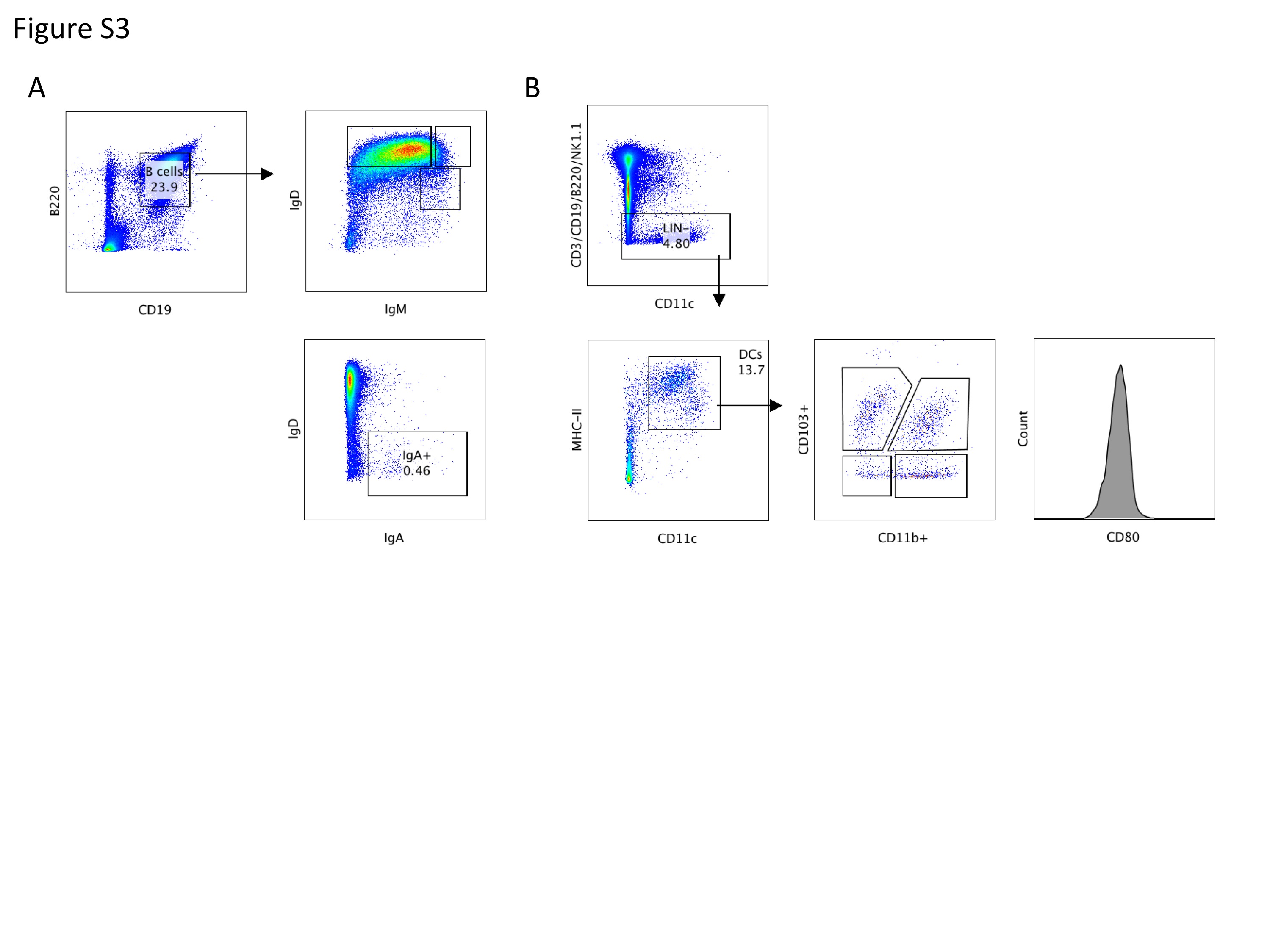

Supplement: Figure S3 — FACS plots B cells and dendritic cells (DCs). Gating strategy and representative FACS plots (mesenteric lymph nodes sample) for identifying B cell and DC subsets. (A) B cells were identified as CD19+B220+ and further divided in IgDhiIgMlo, IgDhiIgMhi, or IgDloIgMhi. In addition, IgA-producing B cells were identified as IgD−IgA+ (B) DCs were identified as lineage negative (CD3−CD19−B220−NK1.1−) and CD11chiMHC-IIhi. These cells were further divided into four subsets based on the expression of CD11b and CD103. In addition, CD80 expression was assessed for each DC subset. [file image_3.jpeg]

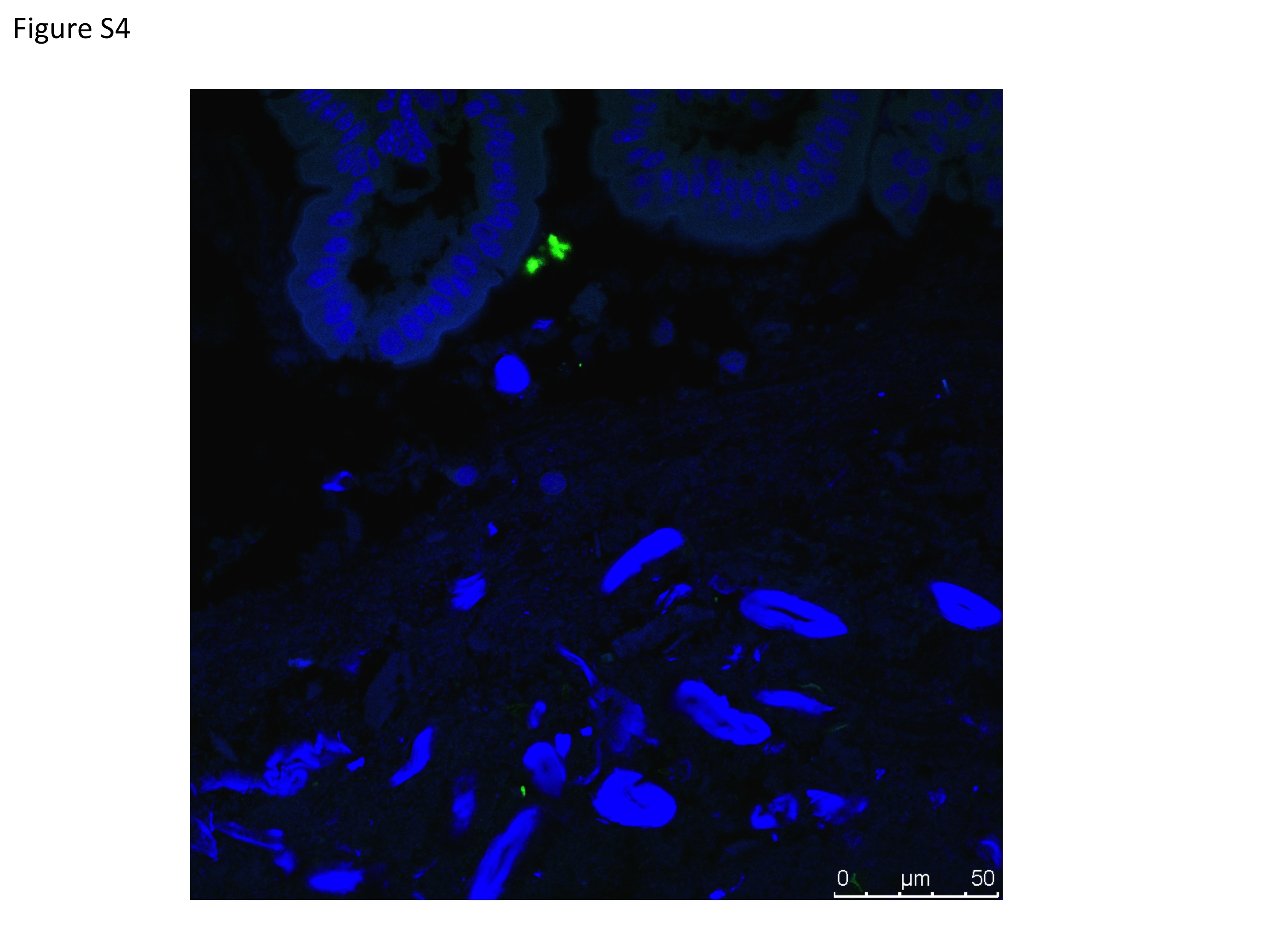

Supplement: Figure S4 — Segmented filamentous bacteria (SFB) FISH staining. A representative image (one mouse treated with short-chain β2→1-fructans) of ileum samples fixed in Carnoy’s fixative that were stained with SFB-specifc probe SFB1008 conjugated to Alexa Fluor 488 (green) and DAPI (blue). Some green spots were identified, but they lacked the typical morphology of SFB. [file image_4.jpeg]
